# Perioperative Expectations of Patients Undergoing Gastrointestinal Surgery for Cancer: A Systematic Review

**DOI:** 10.1245/s10434-025-18439-7

**Published:** 2025-10-14

**Authors:** K. E. Kopecky, O. Monton, S. Koti, C. Arbaugh, J. Purchla, M. Bodd, L. Rosman, F. M. Johnston, J. N. Odom

**Affiliations:** 1 Department of Surgery, University of Alabama at Birmingham, Birmingham, AL USA; 2 Department of Surgery, McMaster University, Hamilton, ON Canada; 3 Department of Surgery, Northwell Health NSLIJ, Manhasset, NY USA; 4 Department of Surgery, Stanford Health Care, Stanford, CA USA; 5 Department of Otolaryngology, Stanford Health Care, Stanford, CA USA; 6 Welch Medical Library, School of Medicine, Johns Hopkins University, Baltimore, MD USA; 7 Department of Surgery, Wake Forest Health, Winston-Salem, NC USA; 8 School of Nursing, University of Alabama at Birmingham, Birmingham, AL USA

**Keywords:** Expectations, Cancer surgery, Gastrointestinal, Patient centered, Systematic review

## Abstract

**Background:**

Despite the prevalence of surgical intervention in patients with gastrointestinal cancers, little is known about patients' expectations regarding physical recovery, long-term prognosis, and quality of life following surgery.

**Methods:**

This systematic review followed the PRISMA reporting guidelines. Four electronic databases (MEDLINE, Embase, PsycINFO, and CINAHL) were searched. Studies were included if they reported the perioperative expectations of adult patients undergoing curative-intent GI cancer surgery. Data extracted included aims and study design, participant characteristics, disease and treatment characteristics, and findings related to patient expectations.

**Results:**

In total, 22 studies were included for analysis and provided data on 3605 patients. The majority of studies found that patients were uncertain about what to expect from surgery, which contributed to feelings of distress, anxiety, frustration, and isolation. Patients struggled to distinguish between normal postoperative symptoms and signs of potential complications and expressed a desire for more information about anticipated and unanticipated surgical outcomes. Expectations regarding the length of postoperative convalescence ranged from weeks to years, and most patients overestimated the ability of surgery to achieve cure. Only one study evaluated the impact of an intervention on preoperative expectation development.

**Conclusions:**

Patients undergoing surgery for resectable GI malignancies are frequently unprepared for the challenges of postoperative recovery and life after surgery. Many express a desire for more thorough and realistic information about what to expect. There is a clear need for the development and evaluation of supportive interventions.

**Supplementary Information:**

The online version contains supplementary material available at 10.1245/s10434-025-18439-7.

According to the International Agency for Research on Cancer, gastrointestinal (GI) cancers account for one in every four new cancer diagnoses,^1^ with surgical intervention estimated to be recommended in 32–45 million cancer cases globally each year.^2^ Additionally, approximately 45–65% of hospital admissions for gastric, pancreatic, esophageal, and colorectal cancer are considered suitable for surgical resection.^2^ Despite the large number of GI cancer surgeries performed each year, there is limited understanding of patients' expectations regarding recovery, psychosocial needs, long-term prognosis, and quality of life following curative-intent surgical resection.

Studies of adults undergoing various types of elective surgery (oncologic, vascular, thoracic, urologic, and neurosurgical) have demonstrated that patients are often surprised by persistent postoperative symptoms that negatively affect their overall well-being and ability to engage in valued life activities.^3–8^ For patients undergoing surgical resection for cancer, evidence demonstrates that deviations from expected postoperative recovery can cause significant distress,^9^ including heightened uncertainty, social isolation, and unmet care needs.^7,10^ Hence, understanding how preoperative expectations are formed and ensuring they align with the experience of postoperative recovery may help to mitigate patient distress.

This systematic review aimed to synthesize the scientific literature on patient expectations regarding surgical resection of GI cancers to clarify and elucidate opportunities for patient education and improve the quality of surgical cancer care. We limited our review to studies involving patients undergoing curative-intent GI surgery for cancer, recognizing that the type and extent of operative intervention can significantly influence both expectation development and postoperative experiences. Studies focusing on palliative-intent procedures were excluded, as the goals and decision-making frameworks for curative and palliative-intent surgeries differ substantially. We anticipate that findings from this review will aid in the development of an intervention to align preoperative patient expectations with the realities of life after surgery.

## Methods

This systematic review examined original research characterizing the perioperative expectations of adult patients who either underwent consultation for, or had already received, curative-intent surgery for GI malignancy, including cancers of the esophagus, stomach, small bowel, liver, pancreas, gallbladder, appendix, colon, rectum, and anus. This review was reported in accordance with the Preferred Reporting Items for Systematic Reviews (PRISMA) statement^11^ and was registered with PROSPERO (CRD42024542722).

### Search Strategy

Two authors (KK and OM) and a senior librarian (LR) designed the search strategy. Four electronic databases (MEDLINE, Embase, PsycINFO, and CINAHL) were searched on April 5, 2024, for peer-reviewed articles indexed prior to the search date, with no date restrictions. Additional studies were identified through manual review and citation searching. The electronic search strategy for the MEDLINE database is provided as an example in Supplementary Material, Table A.

### Study Eligibility

Studies were included if they examined the perioperative expectations of adult patients undergoing curative-intent GI cancer surgery. Exclusion criteria were as follows: (1) non-English language studies, (2) pediatric patient populations, (3) inability to separate GI cancer patients from a mixed-malignancy group, (4) more than 40% of participants undergoing surgery for non-cancer conditions, (5) palliative-intent surgical interventions, and (6) studies focusing solely on postoperative experiences without linking them to expectations. Editorials, reviews, opinion pieces, study protocols, and conference abstracts were also excluded.

### Screening and Full-Text Review

The articles identified from the database searches and manual review were uploaded to the cloud-based Covidence systematic review platform,^12^ and duplicate studies were removed. Six authors (KK, OM, CA, SK, MB, and JP) performed title and abstract screening, and three authors (KK, OM, and SK) performed independent full-text review.

### Data Extraction and Analysis

Data were systematically and independently extracted from all included studies by three authors (KK, OM, and SK). Study characteristics extracted included publication information (title, authors, year published, journal, country of origin), aims and study design (objectives, inclusion criteria, methodology, data collection methods, and timing of data collection), participant characteristics (total number of participants, age, race, gender, ethnicity, insurance status, marital status, and educational attainment), disease and treatment characteristics (primary cancer site, TNM [tumor/node/metastasis] stage,^13^ and surgery performed), and findings related to patient expectations.

### Quality Assessment and Levels of Evidence

Study quality was independently assessed by two investigators (KK and SK) using the Critical Appraisal Skills Programme (CASP) checklists for qualitative research and cohort studies.^14,15^ Further details regarding quality assessment are available in the supplementary material.

## Results

### Search Results and Study Characteristics

**Fig. 1:**
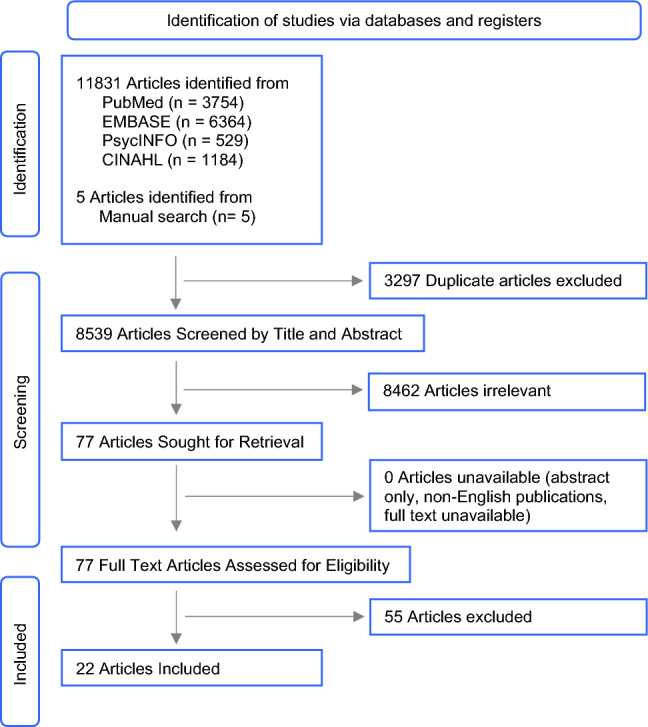
PRISMA diagram. Selection process for study inclusion

A total of 11,831 studies were identified from the four electronic databases, along with five additional studies identified from a manual search. After removing duplicates, 8539 studies underwent title and abstract screening and 77 full-text studies were retrieved and reviewed. A total of 22 studies met our inclusion criteria, were extracted, and are summarized below. The PRISMA flow diagram is outlined in Fig. 1.

Included studies were published from 2010 to 2023 and based in the UK (*n* = 6; 27%), the USA (*n* = 6; 27%), Canada (*n* = 4; 18%), Sweden (*n* = 2; 9%), Belgium (*n* = 1; 5%), Denmark (*n* = 1; 5%), the Netherlands (*n* = 1; 5%), and New Zealand (*n* = 1; 5%). Only four studies (18%) specifically investigated perioperative patient expectations, whereas 18 (82%) identified expectation-related concerns as research findings. Most studies (*n* = 21; 95%) were descriptive/exploratory, and only one study examined the impact of an intervention on the development of patient expectations.^16^ No publications examined the expectations of patients with esophageal, gastric, small bowel, gallbladder, appendiceal, or anal cancers; see Table 1.

**Table 1:** Characteristics of included studies

Characteristics	*n* (%) (*N* = 22)
**Patient population of study**	
Canada	4 (18)
Europe	11 (50)
New Zealand	1 (4.5)
USA	6 (27)
Cancer etiology	
Biliary	1 (5)
Pancreatic	5 (23)
Liver	2 (9)
Colorectal	14 (64)
GI unspecified	2 (9)
**Study design**	
Quantitative	6 (23)
Qualitative	14 (68)
Mixed methods	2 (9)
**Expectations as aims versus findings**	
Primary aim	4 (18)
Secondary aim	2 (9)
Primary finding	7 (32)
Secondary finding	9 (41)
**Method of data collection**	
Focus group	2 (9)
Interview	12 (55)
Survey	6 (27)
Interview and survey	2 (9)
**Timing of data collection**	
Preoperative	3 (14)
Postoperative	15 (68)
< 4 weeks	1 (4.5)
4–12 weeks	5 (23)
> 12 weeks	11 (50)
Unspecified	3 (14)
Both	4 (18)
**Studies that reported demographics**	
Age	22 (100)
Gender	20 (91)
Race	8 (36)
Marital status	7 (32)
Education level	7 (32)
Cancer stage	5 (23)
Insurance status	2 (9)

*GI* gastrointestinal

Of the included studies, 15 (68%) collected data during the postoperative period, three (14%) collected data during the preoperative period, and four (18%) collected data during both the pre- and postoperative periods. Of the included studies, 14 (64%) were qualitative, six (27%) were quantitative, and two (9%) utilized mixed methods. Of the 14 qualitative studies, 12 (86%) utilized interviews and two (14%) gathered data from focus groups.^17,18^ Of the studies that utilized surveys, four (50%) utilized validated and/or standard instruments,^19–22^ and the remaining four relied on investigator-developed (non-validated) surveys.^16,23–25^ Qualitative and mixed methods studies included a median of 21.5 patients (interquartile range [IQR] 8.3), whereas quantitative studies included a median of 47.5 patients (IQR 165.5). Most studies (*n* = 19; 86%) had a sample size of ≤ 50 participants, and three (14%) included > 100 participants. These and other study characteristics are presented in Table 2.

**Table 2:** Included articles and their associated characteristics

Study: article title	Country	Data collection method and timing	Main objective	Total N	Disease etiology	Domains explored	Quality assessment
Abelson et al. ^29^, 2018: Sources of distress among patients undergoing surgery for colorectal cancer: a qualitative study	USA	Pre- and postoperative interview	Explore sources of distress among patients with CRC undergoing surgery	24 (100% surgical)	Colon and rectal	Expectations of physical recovery; psychological experiences as they related to expectations; expectations of social needs	High
Andersson et al. ^26^, 2022: Perceptions of experiences of recovery after pancreaticoduodenectomy - a phenomenographic interview study	Sweden	Postoperative interview	Explore pts’ perceptions of recovery after pancreatic surgery within an ERP	19 (100% surgical)	Pancreatic	Expectations of physical recovery; care at home	High
Beaver et al. ^27^, 2010: An exploratory study of the follow-up care needs of patients treated for colorectal cancer	UK	Postoperative interview	Explore the views of pts on current aspects of their follow-up care	27 (100% surgical)	Colon and rectal	Expectations of physical recovery	High
Brown et al. ^19^, 2013: Colorectal surgery patients' pain status, activities, satisfaction, and beliefs about pain and pain management	Canada	Postoperative survey	Assess postoperative pain and its impact on recovery activities, and pt beliefs and expectations of pain and level of satisfaction of its management	50 (100% surgical)	Colon and rectal	Expectations of physical recovery	Moderate
Burch et al. ^17^, 2023: "You're just on your own": Exploring bowel symptom management needs after rectal cancer surgery through patient and clinician focus groups	UK	Postoperative focus group	Answer the question, “What elements of a bowel management service are needed by people after rectal cancer surgery?”	30 (47% surgical)	Rectal	Expectations of physical recovery; psychological experiences as they related to expectations	High
Burch et al. ^22^, 2023: ‘He's a surgeon, like I'm not going to waste his time’: interviews to determine healthcare needs of people with low anterior resection syndrome after rectal cancer surgery	UK	Postoperative interview and survey	Determine views of pts on their healthcare needs when managing their bowel symptoms after an anterior resection	23 (100% surgical)	Rectal	Expectations of physical recovery	Moderate
Deobald et al. ^38^, 2015: A qualitative study of patient and clinician attitudes regarding surveillance after a resection of pancreatic and peri-ampullary cancer	Canada	Postoperative interview	Determine pt and clinician attitudes towards follow-up after surgical resection of pancreatic adenocarcinoma	22 (68% surgical)	Pancreatic	Prognosis/cure/survival; expectation discordance between pts and providers	High
Harji et al. ^18^, 2015: Development of a conceptual framework of health-related quality of life in locally recurrent rectal cancer	UK	Postoperative focus group	Develop a conceptual model; identify HRQoL issues relevant to pts undergoing surgery for locally recurrent rectal cancer	23 (100% surgical)	Rectal	Prognosis/cure/survival	High
Ibrahim et al. ^30^, 2019: I want to know why and need to be involved in my own care...': a qualitative interview study with liver, bile duct or pancreatic cancer patients about their experiences with involvement in care	Sweden	Postoperative interview	Explore experiences of involvement among pts who had surgery for upper abdominal tumors and were cared for according to a fast-track care program	20 (100% surgical)	Biliary, pancreatic, liver	Psychological experiences as they related to expectations	High
Kim et al. ^20^, 2015: Patient perceptions regarding the likelihood of cure after surgical resection of lung and colorectal cancer	USA	Postoperative survey	Characterize prevalence of expectation that surgical resection of lung or colorectal cancer might be curative	3954 (70% GI)	Colon and rectal	Expectations of physical recovery; prognosis/cure/survival	Moderate
Lafaro et al. ^23^, 2020: Surgeon and patient perceptions of cure in advanced gastrointestinal malignancies: Are we on the same page?	USA	Preoperative survey	Measure correlation of pt and surgeon expectations with perceptions of cure	27 (100% surgical)	Mixed GI, unspecified	Prognosis/cure/survival	Low
McCombie et al. ^35^, 2021: Quality of life preferences in colorectal cancer patients aged 80 and over	New Zealand	Pre- and postoperative interview	Measure and compare what pts with CRC aged ≥80 years and surgeons consider important in terms of survivorship after surgery for CRC	19 (100% surgical)	Colon and rectal	Expectations of physical recovery; prognosis/cure/survival	High
Pape et al. ^28^, 2022: Information and counselling needs of patients with major low anterior resection syndrome: A qualitative study	Belgium	Postoperative interview	Explore information and counselling needs of rectal cancer survivors confronted with major low anterior resection syndrome	28 (100% surgical)	Rectal	Expectations of physical recovery; psychological experiences as they related to expectations	High
Park et al. ^32^, 2014: Patient expectations of functional outcomes after rectal cancer surgery: a qualitative study	Canada/USA	Preoperative interview	Explore pt expectations of outcomes related to bowel function after sphincter-preserving surgery for rectal cancer	26 (100% surgical)	Rectal	Expectations of physical recovery	Moderate
Shinall et al. ^9^, 2023: Patient perspectives on perioperative supportive care needs surrounding major abdominal operations for cancer	USA	Postoperative interview	Determine supportive care needs of pts undergoing major abdominal operations for cancer	47 (100% surgical)	Mixed GI, unspecified	Expectations of physical recovery	Moderate
Spalding et al. ^25^, 2013: Addressing patients' colorectal cancer needs in preoperative education	UK	Pre- and postoperative interview and survey	Understand and develop ways to enhance pts’ experiences of education received before surgery for CRC	138 (70% surgical)	Colon and rectal	Psychological experiences as they related to expectations	High
Streith et al. ^16^, 2022: Effectiveness of a rectal cancer education video on patient expectations	Canada	Preoperative survey	Assess pt expectations of bowel, urinary, and sexual function after rectal cancer treatments, and whether a preoperative education video changed expectations	45 (100% surgical)	Rectal	Expectations of physical recovery	High
Thomsen et al. ^34^, 2017: Patients' vulnerability in follow-up after colorectal cancer: a qualitative action research study	Denmark	Postoperative interview	Identify perspectives of pts undergoing fast-track CRC surgery on challenges experienced in the transition from being a hospitalized pt with cancer to being a cancer survivor	12 (100% surgical)	Colon and rectal	Expectations of physical recovery; psychological experiences as they related to expectations; postoperative surveillance; discharge needs	Moderate
Trobaugh et al. ^21^, 2022: Shared decision-making in pancreatic surgery	USA	Postoperative survey	Evaluate the degree to which pts undergoing pancreatic surgery are involved in the decision-making process, how their recovery aligns with presurgical expectations, and their satisfaction with the decision- making process in relation to quality of life to determine potential areas to intervene to improve SDM	40 (100% surgical)	Pancreatic	Expectations of physical recovery	High
Vandrevala et al. ^37^, 2016: Am I really ready to go home?': a qualitative study of patients' experience of early discharge following an enhanced recovery programme for liver resection surgery	UK	Pre- and postoperative interview	Investigate effect of an ERP on hospital discharge, short- term recovery, and morbidity after open liver resection vs standard perioperative care	20 (100% surgical)	Liver	Psychological experiences as they related to expectations; discharge needs	High
Wancata et al. ^31^, 2022: The patient's perspective: a qualitative study of individual experience with decision-making, treatment, and recovery for resectable pancreatic cancer	USA	Postoperative interview	Gain insight into level of understanding that pts with resectable pancreatic cancer have of their diagnosis, potential treatments, and prognosis	15 (100% surgical)	Pancreatic	Expectations of physical recovery; psychological experiences as they related to expectations; prognosis/cure/survival	High
Wieldraaijer et al. ^24^, 2019: Information needs and information seeking behaviour of patients during follow-up of colorectal cancer in the Netherlands	Netherlands	Postoperative survey	Report on (1) whether pts feel informed about the different subjects of follow-up care and if they need more information on any subject; (2) whether and how pts look for information themselves, and (3) whether there are any differences in subgroups of pts	259 (100% surgical)	Colon and rectal	Postoperative surveillance	High

*CRC* colorectal cancer, *ERP* enhanced recovery program, *GI* gastrointestinal, *HRQoL* health-related quality of life, *pt(s)* patient(s), *SDM* shared decision-making

The included studies reported age in a mix of means and medians, which we expect are roughly equivalent in these patient populations. Among these reported measures, the median patient age across all included studies was 65 years (IQR 8.6). Twenty studies (91%) included information about patient gender, with an average of 52% of patients being male. Eight studies (36%) included data on race and ethnicity. Among the studies that did report demographic information, the average reported patient population was 84% white/Caucasian (range 58–100), 4.6% Black/African American (range 0–14), 3.4% Asian (range 0–13), and 2.9% Hispanic (range 0–15). Five (23%) studies reported information on cancer stage, two (9%) on patient insurance status, seven (32%) on marital status, and seven (32%) on education levels. See Supplementary Material, Table B for further details.

### Quality Assessment

The CASP checklists were used to perform the quality assessment.^14,15^ Study quality ranged from low to high: one (5%) study was rated as low quality, six (27%) studies were rated as moderate quality, and 15 (68%) studies were rated as high quality, see Table 2 and Supplemental Methods.

### Study Findings

Study findings are summarized in Table 3 and described in detail below. Results are grouped in the following seven categories: lack of preoperative preparation and information; sources of preoperative information; expectations of physical recovery; expectations regarding recovery timeline and return to daily life; expectations of postoperative cancer surveillance; expectations regarding prognosis, cure, cancer recurrence, and long-term survival; the impact of preoperative patient education tools on expectation development; and expectations of surgeons to prepare patients for surgery and life after surgery.

**Table 3:** Main findings and representative quotes

Expectation domain	Main findings	Quotes from the manuscripts	Representative pt quotes
Expectations of physical recovery	1. Some pts did not have as many symptoms as they expected, and others experienced more symptoms than they expected	“Unpreparedness resulted in concerns that surgery had gone wrong as well as anxiety about what to do to improve symptoms.”^21^ “Divergences between actual and expected were a source of serious distress.”^9^ “The imminence of [surgery] and concern around [it] prevented some participants from forming expectations related to other outcomes.”^28^	“I was not pre-prepared for the risks … If I had [understood], I would’ve asked more questions, like what the hell is that” (postoperative pt who experienced a complication).^20^
	2. More prominent symptoms were perceived as delaying recovery		“In terms of what my bowel function might be like in the future … I guess I thought that with the surgery it would be normal, so I really hadn’t thought much about it.”^28^
	3. Pts who had inaccurate expectations about physical recovery felt unprepared		“Nobody mentioned anything that your life would never really be the same … this was all quite shocking"^22^
	4. Pts often placed importance on being cancer free and getting through surgery, leaving little time to think about what to expect after discharge from the hospital		“Probably most surprising as I came out of the surgery was … all the tubes and the wires hooked to me.”^9^ “I am pretty sure my bowel function is going to impact me in some way for the rest of my life, but because I know of no other options, I just try to accept it.”^28^
	5. Pts expressed an inability to fully prepare/understand what surgery entailed until they experienced it for themselves		“… there’s no amount of explaining, and once you go through it, oh that’s what that feels like or that’s what they meant, and so I think there’s just some you have to experience …”^27^
Psychological experiences as they related to expectations	1. Included manuscripts describe pt experiences of worry, uncertainty, unpreparedness, anxiety, acceptance, stress, self-blame, hope, fear, loneliness, disappointment, shame, failure, apprehension, frustration, panic, terror, resentment, overwhelm, unsettled, confusion, disempowerment, and vulnerability as related to preoperative expectations, and how their expectations compared with their actual experiences	“The consequence [of not setting postoperative expectations] was that pts felt fear and were unprepared for what happened.”^26^ “[When pts had unanticipated post operative symptoms they] assumed something went wrong during surgery.”^19^ “Information before surgery can help to decrease unrealistic expectations and thus avoid disappointment when confronted with [postoperative symptoms].”^19^	“When you don’t know what to expect, your mind goes reeling in a million different directions and it increases your anxiety.”^20^ “I had moments of fear, anger, panic, resentment that nobody told me how hard it would be.”^20^ “I think I would’ve been much better off had I known that this is just normal to have these complications.”^9^ “I was totally unprepared, it came as a complete surprise … I had wanted to be forewarned.”^26^ “I didn’t realise symptoms might occur so thought things had gone wrong.”^21^ “Nobody mentioned anything that your life would never really be the same … this was all quite shocking”^22^ “If I could have included [what to expect] in my process from the beginning, it would have been very different for me.”^19^
Expectations of prognosis, cure and survival	1. Pts’ understanding and expectations of cancer prognosis contrast with clinicians’ perspectives, even when pts perceived themselves to be well-informed 2. Pts expect that they will be cured after surgery, even if that is not reflective of their clinical situation	“The belief that surgery would be curative among pts with … colorectal cancer was extremely widespread (>80%) and remained high (approximately 60–80%) even among pts with metastatic stage IV disease.”^30^	“I viewed and expected my surgery to be curative and that it is absolute cure, I’m just hoping they have fixed me and it doesn’t come back and I can have a bit longer before I have to worry about it.”^51^ “Hopefully the chemo and radiation will look after that [positive margin and lymph nodes] and get healthy for another 30 years.”^37^ “I would have to do chemo because they see lesions on my liver and they want to get rid of them before they become tumours.”^37^ “I am expecting to be better [after surgery].”^33^

*pt(s)* patient(s)

#### Insufficient Preoperative Preparation and Information

In 15 studies (68%), patients reported feeling unprepared for surgery and postoperative recovery and expressed a need for more preoperative information to better prepare for the challenges they faced after surgery.^17,22,26–29^ In a survey study of 40 patients undergoing pancreatic surgery, Trobaugh et al.^21^ examined the relationship between preoperative expectations and the experience of recovery. Thirteen respondents (32.5%) felt that changes in postoperative overall quality of life were not well explained.^12,21^ Similarly, Wieldraaijer et al.^24^ surveyed 259 patients who had undergone curative-intent surgery for colorectal cancer and found that 49% were unsure of what to expect in the future, 43% felt uninformed about nutritional needs, and 36% had questions about how to manage postoperative symptoms. In this study, 33% of survey respondents had searched for information on their own to learn more, primarily by searching the internet, by asking friends, or by reading an informational brochure.^24^ Additionally, two separate studies of patients who had undergone surgery for rectal cancer found that, although patients perceived the preoperative information as sufficient, they faced significant challenges when there was discordance between their expectations and actual experiences.^18,28^ One-third of the included studies reported that being unprepared for surgery and recovery was a significant source of patient distress.^9,17,28,29^ Patients reported feelings of anxiety, anger, panic, frustration, and resentment when faced with unanticipated challenges.^17,28–31^

#### Sources of Preoperative Information

Six studies (27%) commented on the influence of preoperative information-seeking behavior and patient expectation development.^9,17,28,30,32,33^ Patients relied on written material, online content, and other sources of preoperative information such as primary medical literature, second surgical opinions, and interactions with online peer-to-peer support groups and message boards to learn about what to expect from surgery.^32^ Three studies (17%) commented on the potential for these sources to offer unreliable information.^9,17,32^ Abelson et al.^29^ found that sometimes too much operative information sharing by surgeons and surgical staff contributed to increased stress and worry. Similarly, Burch et al.^17^ hypothesized that “information overload” might contribute to patient unpreparedness if it led to feelings of overwhelm and decreased motivation to seek information about what to expect.

#### Expectations of Physical Recovery

Fifteen studies (68%) examined expectations related to physical recovery, including symptoms such as pain, postoperative bowel, urinary, and sexual dysfunction, mobility, fatigue, activity restrictions, changes in sleep habits, and the management of surgical complications.^9,16,17,19–22,26–29,31,32,34,35^ The included manuscripts also described patient experiences of worry, uncertainty, unpreparedness, anxiety, and acceptance regarding the physical changes resulting from surgery.^9,17,22,27,29,31,32^

### Expectations of Postoperative Hospitalization and Inpatient Monitoring

Only one study (5%) explored patient expectations regarding the immediate postoperative period, including in-hospital recovery.^9^ Shinall et al.^9^ reported that patients undergoing major abdominal surgery for cancer often felt surprised, overwhelmed, and afraid to be connected to medical devices, such as nasal cannula, nasogastric tubes, surgical drains, and Foley catheters.

### Expectations of Postoperative Pain

Five studies (23%) examined expectations of postoperative pain.^9,16,19,29,31^ In their survey of 50 postoperative patients who had undergone surgery for colorectal cancer, Brown et al.^19^ found that most patients expected to experience pain after surgery. Abelson et al.^29^ noted that patients who were preparing to undergo surgery for colorectal cancer experienced excessive worry and preoccupation about the uncertainty of postoperative pain. Shinall et al.^9^ authored the only study that explored non-incisional pain, such as pain from abdominal bloating, musculoskeletal soreness, or an infiltrated intravenous line. These pain experiences were reported as devastating, surprising, frightening, and concerning by a mixed patient population who had undergone major abdominal surgery for cancer.^9^ Lastly, Wancata et al.^31^ found that some patients reported that intellectually understanding that there would be pain after surgery was very different from actually experiencing it.

### Expectations of Changes in Appetite, Oral Intake, and Upper GI Symptoms

Seven studies (31%) examined patient expectations of changes in appetite, oral intake, and upper GI symptoms.^17,21,25,27,28,31,34^ Beaver et al.^27^ interviewed 41 patients who had undergone colorectal cancer surgery. Patients in this study reported receiving little to no guidance on recommended postoperative dietary changes to help manage stool urgency, consistency, and the impact of postoperative oral intake on bowel function.^27^ Thomsen and Hølge-Hazelton^34^ found that patients were unprepared for appetite loss. Similar findings of unanticipated upper GI symptoms were reported by others.^21,25,28,31^ Patients across multiple studies expressed an expectation that they should have received dietary guidance before surgery.^17,27^ Because patients were unaware of the need for postoperative dietary adjustments, many relied on a trial-and-error approach, which resulted in anxiety.^17,27,28^

### Expectations Regarding Changes to Bowel Habits and Postoperative Bowel Dysfunction

Participants in 10 studies (45%) reported being unprepared for persistent changes in bowel habits.^17,18,22,26–29,31,32,34^ One study in patients undergoing colorectal cancer surgery found this lack of preparedness to be associated with distress, particularly when postoperative experiences did not align with preoperative expectations.^29^ Patients in this study also expressed worry, anxiety, and sadness about the possibility or necessity of living with a temporary or permanent stoma.^29^ Furthermore, three studies reported that patients who had undergone colorectal cancer surgery felt unprepared for the increased frequency and urgency of bowel movements, changes in stool consistency, increased flatulence, and pain associated with passing stool after surgery.^17,18,29^ Many patients in the study by Burch et al.^17^ reported a lack of understanding regarding how long postoperative changes in bowel habits would persist. In four studies, patients described the process of adjusting to a “new normal” in their bowel habits, often accompanied by the unsettling realization that their lives (and bowel function) would never fully return to their preoperative state. This was not something they had anticipated.^22,26,31,34^ These unanticipated changes in bowel patterns had further negative consequences on patients’ sleep, work, social activities, and ability to leave the house.^22^ Patients felt that being able to prepare for these changes would have helped mitigate the distress associated with dealing with them unexpectedly after surgery.^17,22^ In one study of patients scheduled for rectal cancer surgery, the authors identified three key findings related to preoperative expectations regarding postoperative bowel function: (1) general uncertainty about the degree of postoperative bowel dysfunction, (2) a prioritization of survival outcomes over functional bowel outcomes, and (3) the perception that surviving surgery was a more pressing worry than navigating survivorship.^32^ These themes were also reflected in other studies.^9,17,28^ Many patients experienced shame, embarrassment, and insecurity in managing their bowel function and expected these concerns to be proactively addressed but often found that they were not.^18,28^ Similarly, patients across multiple studies expressed a clear expectation that they should have been informed about expected postoperative bowel dysfunction prior to agreeing to surgery.^22,28,30^

### Expectations Regarding the Side Effects of Surgery and Possibility of Complications

Five studies (23%) examined patient expectations regarding side effects of surgery and the possibility of complications.^9,18,20,29,30^ Kim et al.^20^ collected survey data from 2755 patients and examined their perceptions of symptom relief and complications following colorectal cancer surgery. They found that 45% of patients with colorectal cancer indicated that they believed surgery was “somewhat likely” or “very likely” to contribute to side effects and/or postoperative complications.^20^ Ibrahim et al.^30^ performed postoperative qualitative interviews and characterized the expectations of patients who experienced postoperative complications or side effects following surgery for hepatobiliary cancer. The authors found that patients felt fearful, surprised, and unprepared when they experienced side effects that, although uncommon, were not unexpected—such as developing diabetes after pancreatic resection. This finding was similar to that of Shinall et al.^9^ and Abelson et al.^29^, who conducted semi-structured interviews with patients who had undergone abdominal surgery for cancer and found that interview participants who did experience complications felt unprepared and wished they would have received more information. Finally, Harji et al.^36^ found that patients who had undergone surgery for locally recurrent rectal cancer expressed frustration with the development of unanticipated postoperative outcomes, such as issues with wound healing, fistula formation, postoperative intestinal obstruction, and the development of incisional hernias.

### Lack of Clarity Regarding Normal Versus Concerning Postoperative Symptoms

In nine studies (41%), patients reported not understanding whether their physical symptoms were “normal” for the type of surgery they had undergone.^9,17,22,26–29,34,36^ Many patients misinterpreted routine but unexpected postoperative symptoms as signs of a surgical complication^17,27,28^ or cancer recurrence.^18,22,26,28^ When patients were not prepared for these outcomes, these experiences contributed to feelings of fear, loneliness, vulnerability, and isolation.^17,28,31,34^

#### Expectations Regarding the Timing of Recovery and Return to Daily Life

Two studies (9%) examined patient expectations of hospital length of stay,^26,36^ three studies (14%) specifically reported on patient discharge needs and expectations,^27,30,37^ and three studies (14%) commented on patient expectations of the recovery.^17,26,36^

### Expectations Regarding Hospital Length of Stay and Discharge

A qualitative study of patients undergoing surgery for locally recurrent rectal cancer found that patients did not anticipate extended hospital stays.^18^ Conversely, Andersson et al.^26^ reported mixed expectations regarding hospital length of stay, with some patients expecting a longer stay and others expecting a shorter stay. In some cases, discharge signaled to patients that their recovery was progressing as expected.^37^ Beaver et al.^27^ noted that many patients were unsure of what to expect when they were released from the hospital. Similarly, Ibrahim et al.^30^ found that patients who had been prepared for discharge felt safe, whereas others who had not expected to be discharged felt anxious, neglected, and afraid.

### Expectations of the Timing of Recovery

A qualitative study of 19 patients with pancreatic cancer reported a wide range of expectations regarding the length of postoperative recovery at home following discharge from hospital, with some patients expecting postoperative recovery to be completed within weeks, and others anticipating that it might take years.^26^ In contrast, patients with rectal cancer reported that they expected a faster postoperative recovery than what they actually experienced.^17,36^

### Expectations of Return to Daily Life

Nine studies (41%) found that surgery disrupted patients’ daily routines in unexpected ways. Some patients assumed life would return to normal after surgery, and others were uncertain about what to expect postoperatively.^26,29,37^ Several studies reported that surgery unexpectedly and negatively impacted independence in self-care activities such as toileting, cooking, and laundry, as well as the ability to work, leave home, drive, shop for groceries, maintain a lawn, complete household chores, and participate in social activities.^17,18,25,26,29^ Early postoperative fatigue, along with persistent fatigue and decreased energy levels, significantly affected patients’ ability to carry out tasks they once considered routine.^18,31,34^ Patients interviewed postoperatively described the challenge of accepting a “new normal” and noted that recovery took longer than expected.^27,29,34^

#### Expectations of Postoperative Cancer Surveillance

Three studies (14%) reported on patient expectations regarding postoperative surveillance.^24,34,38^ Two studies (9%) focused on patients who had undergone colorectal surgery and participated in early recovery after surgery programs or hospital-based follow-up programs. In these studies, participants were aware that they would be monitored for cancer recurrence postoperatively.^24,34^ Deobald et al.^38^ conducted qualitative interviews with 15 patients who had undergone surgical resection of pancreatic or periampullary adenocarcinoma and were undergoing surveillance. These patients expected that frequent imaging studies would detect potential tumor recurrence earlier, thereby improving their overall survival.^38^ No other studies mentioned or addressed patient expectations or knowledge of postoperative surveillance programs.

#### Expectations Regarding Prognosis, Cure, Recurrence, and Long-Term Survival

Six studies (27%), two quantitative and four qualitative, reported patient expectations of cancer cure and/or recurrence.^20,23,31,35,36,38^ Overall, patients expected that surgery would eliminate their cancer, and a majority of patients overestimated the ability of surgery to achieve cure.^20,38^ Though it was not consistently stated as an expectation, participants across several studies reported hopefulness and optimism associated with surgery for cancer.^18,31,32,37^

### Expectations Regarding Postoperative Prognosis and Cure

Four studies (18%) examined patients’ expectations regarding postoperative prognosis and cure.^18,20,23,38^ Overall, patients viewed themselves as being cancer free after surgery and had a limited understanding of their overall prognosis. Kim et al.^20^ examined patient perceptions regarding the likelihood of cure following surgical resection of colorectal cancer. The researchers found that 90.5% of patients with stage III colorectal cancer expected that surgery was likely to cure their cancer. Underrepresented racial and ethnic groups (Hispanic, African American, or Asian American), higher income, and higher educational attainment were associated with a greater perception that surgery would offer cure (all *p* < 0.05).

### Expectations Regarding Cancer Recurrence

Three studies (14%) explored patient expectations of postoperative cancer recurrence.^18,31,38^ Harji et al.^18^ conducted focus groups including 23 adult patients after surgical resection for locally recurrent rectal cancer. The researchers found that patients experienced surprise, shock, and disappointment upon receiving a cancer recurrence diagnosis, indicating that it was unexpected.^18^ Wancata et al.^31^ and Deobald et al.^38^ conducted postoperative qualitative interviews with patients after surgical resection of pancreatic adenocarcinoma, including nine patients who had experienced recurrence at the time of the interview. Among the patients who did not experience recurrence, many held onto hope that they might “be one of the ones who make it”.^31^ Unlike patients with colorectal cancer, those with pancreatic cancer had a wider range of expectations. Some acknowledged their poor prognosis and the high recurrence rates of pancreatic cancer,^31^ whereas others had unrealistic expectations about overall survival, including some who expected to live an additional 10 years or more.^38^

### Discordant Expectations Between Patients and Providers

Two publications (9%) reported on discordant expectations between patients and providers.^23,38^ Deobald et al.^38^ conducted qualitative interviews with patients who underwent surgical resection for pancreatic adenocarcinoma. All clinicians in this study reported informing patients about the risk of recurrence. However, researchers identified a “major disconnect” between patients and surgeons in understanding the likelihood of recurrence and overall cancer prognosis, including patients’ unrealistic survival expectations.^38^ Similarly, Lafaro et al.^23^ conducted a survey to assess patients' preoperative perceptions of their postoperative prognosis and likelihood of cure among patients undergoing GI cancer surgery. They found that 71% of patients expected surgery to cure their cancer, and over 80% believed they would survive beyond 5 years. In contrast, surgeons estimated that only 32% of patients would be alive at 5 years.

#### Impact of Preoperative Patient Education Tools on Expectation Development

Only one study (5%) evaluated the impact of an intervention on preoperative expectation development.^16^ This single-center prospective cohort study enrolled 45 patients and evaluated the effectiveness of a rectal cancer education video on patient expectations regarding postoperative anorectal function.^16^ Data were collected via pre- and postintervention survey administration. The authors found that expectations regarding postoperative bowel control changed in 44–69% of patients who watched a preoperative informational video, whereas expectations regarding urinary and sexual function remained unchanged. Specifically, patients who watched the video were more likely to expect that they would require medications for bowel control (*p* = 0.03), that they might need to rush to the toilet (*p* = 0.06), and that they might need to make alterations to their diet for the purposes of bowel control (*p* = 0.10).^16^

#### Patients Expect Surgeons to Prepare Them for Surgery and Life After Surgery

In one study (5%), patients emphasized their expectation that surgeons should provide information directly, rather than requiring them to figure things out on their own.^28^ Overall, patients wanted more information about what to expect after surgery and to receive postoperative symptom support.^9,22,24,28^ Their expectations for information extended beyond the immediate perioperative period; they also wanted to be prepared for life after surgery, survivorship, and changes to daily habits.^28^ Patients reported an expectation of being informed about possible postoperative outcomes, even if discussing such information was difficult or could have caused additional stress.^28^

## Discussion

This systematic review assessed studies characterizing the expectations of patients undergoing surgery for GI cancer. A total of 22 studies met our inclusion criteria, including 14 qualitative studies, six quantitative studies, and two mixed-methods studies. Only one study specifically evaluated how a patient-directed educational intervention influenced preoperative expectations.^16^ Overall, the included studies revealed that patients often overestimated the curative potential of surgery and underestimated their need for information and support. This expectation gap led patients to feel frustrated, distressed, and uncertain. Though not all patients have a defined set of postoperative expectations regarding their physical recovery, they have deeply rooted expectations about the care and information they receive from the surgical team.

The lack of well-developed and informed expectations is likely driven by multiple factors. First, surgeons are not routinely trained in communication^39–41^ and often use language that drastically oversimplifies the experience of surgery and surgical recovery while also failing to establish the goal of treatment.^42^ This, coupled with patient cognitive and emotional overwhelm when processing difficult diagnoses and complex medical information,^43–46^ may lead to misunderstanding and increased distress in the perioperative period. Additionally, surgeons can be hesitant to share potentially distressing details, such as honest prognostic assessments, and may struggle to find the right balance between providing too little and too much information.

### Future Directions in Research and Clinical Care

As our understanding of what matters most to patients continues to evolve, particularly through the incorporation of patient-reported and patient-centered outcomes into prospective trials, it will become increasingly evident that a more comprehensive approach to perioperative expectation management is required. The surgical community has the opportunity to equip patients with the information and emotional support they need to feel adequately prepared for life after surgery^47–49^ while also informing patients about the range of possible postoperative outcomes, including scenarios in which things do not proceed as expected. Additional research is needed to better understand how patients seek and retain information about what to expect in the postoperative setting, the best methods for helping patients manage their expectations, and predictors of misaligned expectations. In addition, future research should incorporate rigorously collected quantitative data to complement qualitative insights and help characterize the prevalence, variation, and impact of patient expectations across diverse populations. Additionally, there are currently no validated tools to measure expectations; future research should be conducted to reliably measure and monitor expectations in the context of clinical cancer care. Building on this, additional areas of future research include examining how socioeconomic status, educational attainment, and peer-to-peer interactions influence the development of perioperative expectations, as well as exploring the relationship between expectation alignment and postoperative regret. Finally, future prospective interventional trials designed to align patient expectations with the experience of surgical recovery will offer an opportunity to learn more about which preoperative educational elements have the greatest impact on expectation development, as well as our ability to modify expectation development in the clinical setting.

### Limitations

This study has several limitations. Although the review was conducted systematically, there remains a possibility that relevant literature may have been overlooked. We made every effort to include studies that specifically focused on patient expectations within our target population, as well as those in which expectations were identified as a key factor or finding. Notably, most of the included studies were descriptive and involved small sample sizes (*n* < 50) with variable quality. Although this review focused exclusively on patients with GI malignancy, it is important to note that these cancers encompass a broad spectrum of postoperative outcomes and prognoses, which may limit the generalizability of our findings. Lastly, comparison between studies is limited by the lack of a standardized tool to capture and assess patients’ expectations.^50^

## Conclusion

This systematic review reveals that patients undergoing surgery for resectable GI malignancy are often unprepared for the realities of postoperative recovery and life after surgery. Patients report limited preoperative understanding of the recovery process and struggle to differentiate between expected postoperative challenges and complications. Patients consistently express a need for more comprehensive information about what to expect after GI cancer surgery, and they look to their surgical teams to not only prepare them for the long-term consequences of surgery but also provide continued guidance and symptom support.

## Supplementary Information

Below is the link to the electronic supplementary material.

Supplementary file 1 (DOCX 46 kb)
