# Supplementary material for: Perioperative Expectations of Patients Undergoing Gastrointestinal Surgery for Cancer: A Systematic Review
Source: Ann Surg Oncol. 2025 Oct 14;33(2):1417–32. doi: 10.1245/s10434-025-18439-7 (PMC12765745; doi:10.1245/s10434-025-18439-7)
Supplement: Supplementary file 1 — (DOCX 46 kb) [file 10434_2025_18439_MOESM1_ESM.docx]

**Perioperative Expectations of Patients Undergoing Gastrointestinal Surgery for Cancer: A Systematic Review**

**Authors:** Kopecky KE^1^, Monton O^2^, Koti S^3^, Arbaugh C^4^, Purchla J^3^ Bodd M^5^, Rosman L^6^, Johnston FM^7^, Odom, JN ^8^

**Supplemental Material**

**Table A.** Medline (Ovid) search strategy.

| 1 | **exp "Qualitative Research"/ or exp "Surveys and Questionnaires"/ or exp "Psychometrics"/ or exp "Focus Groups"/ or exp "Interview, Psychological"/ or exp "Grounded Theory"/ or exp "Interviews as Topic"/ or exp "Interview"/ or exp "Narration"/ or exp "Self Disclosure"/ or exp "Disclosure"/ or exp "Needs Assessment"/ or exp "Self-Assessment"/ or exp "Sickness Impact Profile"/ or exp "Patient Outcome Assessment"/** |
| --- | --- |
| 2 | (qualitative* or survey or surveys or surveyed or questionnaire* or psychometric* or "focus group*" or interview* or "deliberative forum*" or "open ended" or "semi structured" or "grounded theor*" or "content analys*" or "discourse analys*" or "framework analys*" or "mixed method*" or "needs assess*" or "One-on-one" or Inductive).tw,kf. |
| 3 | 1 or 2 |
| 4 | **exp "Neoplasms"/** |
| 5 | (neoplas* or cancer* or tumor* or tumour* or malign* or oncolog* or carcinoma*).tw,kf. |
| 6 | 4 or 5 |
| 7 | **(exp "Digestive System Surgical Procedures"/ or exp "Colorectal Surgery"/)** |
| 8 | (gastrectom* or pyloromyotom* or cholecystectom* or enterectom* or colectom* or hemicolectom* or proctocolectom* or hepatectom* or pancreatectom* or proctectom* or esophagectom*).tw,kf. |
| 9 | **(exp "Surgical Procedures, Operative"/ or exp "General Surgery"/ or exp "Surgical Oncology"/) and (exp "Digestive System"/)** |
| 10 | ((abdom* or gastr* or stomach* or gallbladder* or intestin* or colon* or colorectal* or bowel* or liver* or "bile duct*" or pancrea* or hepat* or biliar* or rectal* or rectum* or anus or anal) and (surg* or operat* or resect* or excision* or removal* or cytoreduct* or perioperativ* or peri-operativ* or postoperativ* or post-operativ* or bypass)).tw,kf. |
| 11 | or/7-10 |
| 12 | **(exp "Patients"/ or exp "Caregivers"/ or exp "Family"/) and (exp "Uncertainty"/ or exp "Comprehension"/ or exp "Anticipation, Psychological"/ or exp "hope"/ or exp "Psychological Distress"/ or exp "anxiety"/ or exp "Stress, Psychological"/ or exp "Perception"/ or exp "Narrative Medicine"/ or exp "Attitude"/)** |
| 13 | ((patient* or individual* or self or subject* or participant* or person* or family or families or carer* or parent* or father* or mother* or spouse* or husband* or wife or wives* or child* or sibling* or relative* or partner or partners) **adj4** (expect* or anticipation* or hope or hopes or uncertaint* or comprehension or understanding* or distress* or anxiet* or stress* or mismatch* or report* or perception* or perspective* or experience* or descript* or describe* or narrative* or narration or burden* or attitude* or opinion* or accept*)).tw,kf. |
| 14 | 12 or 13 |
| 15 | 3 and 6 and 11 and 14 |
| 16 | Limit 15 to english language |

**Table B.** Reported Patient Demographics by Study

| **First Author/ Year Published** | **Patient Age** | **Male** | **Patient Race/Ethnicity** | **Insured** | **Married** | **Patient Education** | **Cancer Stage** |
| --- | --- | --- | --- | --- | --- | --- | --- |
| Abelson 2018 | 63 (mean) | 8 (33%) | 18 (75%) White  3 (13%) Black  2 (8%) Asian  2 (8%) Hispanic | -- | -- | -- | 6 (25%) Stage I  9 (38%) Stage II  8 (33%) Stage III  1 (4%) Stage IV |
| Andersson 2022 | 66 (mean) | -- | -- | -- | -- | -- | -- |
| Beaver 2010 | 72 (mean) | 14 (52%) | 27 (100%) White | -- | 20 (74.1%) | -- | -- |
| Brown 2013 | 67 (mean) | 27 (54%) | -- | -- | -- | -- | -- |
| Burch 2023 | 55 (mean) | 7 (50%) | -- | -- | -- | -- | -- |
| Burch 2023 | 53 (median) | 10 (43%) | -- | -- | -- | -- | -- |
| Deobald 2015 | 64 (median) | 8 (53%) | 13 (87%) White | -- | 11 (73%) | 7 (32%) High School  6 (27%) Undergraduate  2 (9%) Graduate | -- |
| Harji 2015 | 63 (median) | 12 (52%) | -- | -- | -- | -- | -- |
| Ibrahim 2019 | 68 (mean) | 10 (50%) | -- | -- | -- | -- | -- |
| Kim 2015 | 65 (median) | 1351 (49%) | 1161 (58%) White  377 (14%) Black  151 (5%) Asian  211 (8%) Hispanic  120 (4%) Other | 2393 (87%) | 1589 (58%) | 398 (14%) High school  1356 (49%) High school degree or some college  715 (26%) College degree | 2435 (61.6%) Stage I/II  1023 (25.9%) Stage III  496 (12.5%) Stage IV |
| Lafaro 2020 | 60 (median) | -- | 16 (60%) While  2 (7%) Black  2 (7%) Asian  4 (15%) Hispanic | -- | -- | 5 (19%) High school  14 (52%) Some College  3 (11%) Completed College  Beyond college 5 (19%) | -- |
| McCombie 2021 | 88 (median) | 8 (42.1%) | -- | -- | -- | -- | 6 (31.6%) Stage I  8 (42.1%) Stage II  8 (42.1%) Stage III  2 (10.5%) Stage IV |
| Pape 2022 | 54 (mean) | 18 (64%) | -- | -- | -- | -- | -- |
| Park 2014 | 56 (mean) | 14 (54%) | -- | -- | 14 (53.8%) | -- | 6 (23.1%) Stage 0  7 (26.9%) Stage I  6 (23.2) Stage II  7 (26.9%) Stage III |
| Shinall 2023 | 64 (median) | 24 (51%) | 46 (98%) White  1 (2%) Black | -- | -- | 10 (21%) High school  29 (62%) College  8 (17%) Graduate degree | -- |
| Spalding 2013 | 70 (mean) | 55 (56%) | -- | -- | -- | -- | -- |
| Streith 2022 | 58 (mean) | 34 (75%) | -- | -- | -- | 4 (8.9%) < High school  13 (28.9%) High school  13 (28.9%) College degree  2 (4.4%) Graduate degree | -- |
| Thomsen 2017 | 72 (mean) | 5 (42%) | -- | -- | 9 (75%) | -- | -- |
| Trobaugh 2022 | 66 (mean) | 15 (37.5) | 38 (95%) White  1 (3%) Black  1 (3%) Asian | 40 (100%) | -- | 14 (35) High school  9 (22.5) 2 yr college  17 (42.5) 4 y college | -- |
| Vandrevala 2016 | 63 (mean) | 12 (60%) | -- | -- | -- | -- | -- |
| Wancata 2022 | 66 (mean) | 9 (60%) | 15 (100%) White | -- | -- | -- | -- |
| Wieldraaijer 2019 | 67 (mean) | 141 (54%) | -- | -- | 198 (76%)  Living together  61 (24%)  Living alone | 12 (5%) < High school  149 (58%) High school  70 (27%) Vocational  28 (11%) University | 73 (28%) Stage I  88 (34%) Stage II  98 (38%) Stage III |

* -- denotes unavailable data

**Table C.** Reported Patient Demographics

|  |  |
| --- | --- |
| **Population Characteristics**  Average age (years)  % Male (average)  Race/Ethnicity  % White  % Black  % Asian  % Hispanic  % Other  Marital Status  % Married | 64 *(n=22)*  52% *(n=20)*  *(n=8)*  84%  4.6%  3.4%  2.9%  0.5%  *(n=7)*  69% |

**Supplemental Methods for Quality Assessment and Levels of Evidence**

Study quality was independently assessed by two investigators using the Critical Appraisal Skills Programme (CASP) checklists for qualitative research and cohort studies.^14,15^ Further details can be found in the supplementary content. The qualitative checklist consists of 10 Yes/No questions that evaluate a study across three domains: (1) validity of study results, (2) ethics and rigor of the study design, and (3) contribution of the research to the field. For this review, qualitative studies were classified as: High Quality when all ten questions were answered ‘yes’ and the research was considered valuable to the field; Moderate Quality if 7-9 questions were answered ‘yes;’ Low Quality if 4-6 questions were answered ‘yes;’ and Very Low Quality if less than 4 questions were answered ‘yes.’ The cohort study checklist consists of 12 questions covering the same domains. For this review, cohort studies were classified as: High Quality if 12 questions were answered ‘yes;’ Moderate Quality if 9-11 questions were answered ‘yes;’ Low Quality if 4-8 questions were answered ‘yes;’ and Very Low Quality if less than 5 questions were answered ‘yes.’
